# Moving Forward Together: A Protocol to Co-Adapt and Scale a Videoconference-Delivered Physical Activity Intervention for Children and Adolescents Diagnosed With Cancer or Blood Disorders in British Columbia, Ontario, and the Maritime Provinces

**DOI:** 10.2196/92574

**Published:** 2026-05-29

**Authors:** Amanda Wurz, Lauren Ha, Vanessa Sales, Djellza Dani, Caron Strahlendorf, Kristin Marr, Hanna Lotocka-Reysner, Ewa Lunaczek-Motyka, Anne Carrelli, Brianna Empringham, Raveena Ramphal, Donald Mabbott, Chelsea Ash, Annette Flanders, Mary Stuart, Christopher Consmueller, Melanie R Keats, Emma McLaughlin, S. Nicole Culos-Reed, Gregory MT Guilcher, Sara Fisher, Carolina Chamorro-Viña, IMPACT Co-Adaptation Advisory Board Members & Research Program Partners and Collaborators

**Affiliations:** 1 University of the Fraser Valley Chilliwack, BC Canada; 2 Jewish General Hospital Montreal, QC Canada; 3 BC Children's Hospital Vancouver, BC Canada; 4 Surrey Memorial Hospital Surrey, BC Canada; 5 Victoria General Hospital Victoria, BC Canada; 6 Children's Hospital of Eastern Ontario Ottawa, ON Canada; 7 Hospital for Sick Children Toronto, ON Canada; 8 Izaak Walton Killam Health Centre Halifax, NS Canada; 9 Dalhousie University Halifax, NS Canada; 10 University of Calgary Calgary, AB Canada; 11 Alberta Children's Hospital Calgary, AB Canada; 12 Stollery Children's Hospital Edmonton, AB Canada; 13 Kids Cancer Care Foundation of Alberta Calgary, AB Canada

**Keywords:** exercise, movement, pediatric oncology, neoplasms, internet-based, online, remote, implementation, co-design, partnered approach, patient engagement

## Abstract

**Background:**

Physical activity (PA) is safe and beneficial for children and adolescents diagnosed with cancer, yet most engage in low levels of PA. We developed IMPACT (IMplementation of Physical Activity for Children and adolescents on Treatment), a PA intervention delivered by videoconference to enhance PA among young people during treatment for cancer and blood disorder diagnoses. IMPACT is being evaluated in a type II hybrid effectiveness-implementation trial in Alberta, Canada. While referral rates are high and early visual analyses suggest IMPACT may enhance PA and aspects of quality of life and physical function, participation, retention, and adherence rates are low. Findings signal the positive effect of IMPACT for those who participate and underscore the necessity of implementation adaptations. On the basis of these early findings, a demonstrated desire, and funding for PA at sites across Canada, we must first reimagine IMPACT through active collaboration with research users–those who will refer to and/or use or benefit from the intervention.

**Objective:**

Over the next 5 years, our larger research program will (1) co-adapt IMPACT and prepare for scaling (phase 1) and (2) implement and evaluate co-adapted IMPACT across additional provinces in Canada (phase 2). Specific aims for phase 1 are detailed herein and include (1) identifying necessary IMPACT modifications, (2) examining site-specific factors influencing IMPACT implementation, and (3) developing an implementation research logic model to guide continued scaling.

**Methods:**

An integrated knowledge translation and patient-oriented research approach and pragmatic orientation have been adopted. A multiple-perspective mixed methods study is underway. Descriptive surveys and interviews, guided by the Consolidated Framework for Implementation Research 2.0, are being conducted with key research user groups, including children and adolescents diagnosed with cancer and blood disorders (on- and off-treatment), carers, health care providers, and support organization personnel. Data will be analyzed using descriptive statistics and framework analysis. An implementation research logic model will be developed with participants and IMPACT co-adaptation advisory board members and program partners and collaborators.

**Results:**

Funding was secured, and initial ethics approval was granted on June 10, 2025. Additional administrative and full approvals were secured subsequently. Recruitment started in July 2025 in British Columbia and is commencing across sites in a staggered manner. Full results (ie, all site-specific modifications and implementation strategies and the final version of the implementation research logic model) are expected to be submitted for publication late 2026.

**Conclusions:**

Co-adaptation of IMPACT with research users will enhance the likelihood of relevance, acceptability, and uptake nationally. The resulting data will inform a model to guide continued scaling and a larger trial evaluating the co-adapted IMPACT intervention across British Columbia, Ontario, and the Maritime provinces. This work reimagines IMPACT for broader applicability across varied Canadian contexts..

**International Registered Report Identifier (IRRID):**

DERR1-10.2196/92574

## Introduction

There are more than 1500 new diagnoses of cancer in children and adolescents in Canada each year [1]. During treatment, children and adolescents commonly experience symptoms, such as neuropathy, nausea, fatigue, muscle weakness, decreased functional capacity, anxiety, and depression [2-7]. Nearly 85% of those diagnosed will survive 5 or more years after treatment completion [8]; however, survival does not guarantee a return to one’s prior health and functional status [9]. Once completed treatment, young people face increased risks of physical, psychological, social, and cognitive impairments that can reduce quality and length of life [10-16]. Indeed, the physical burden is considerable, with markedly increased risks of secondary cancers (15 times), morbidity (3-54 times), and premature mortality (7 times) compared with noncancer controls [17-24]. Emotional, social, and cognitive difficulties are also common [25,26]. These challenges emphasize a need for strategies that can improve health outcomes in both the short- and long-term for children and adolescents diagnosed with cancer [27].

One strategy that may protect and enhance physical, psychological, social, and cognitive outcomes shortly after diagnosis is physical activity (PA). PA is defined as any movement produced by skeletal muscles that results in energy expenditure [28]. Synthesized experimental evidence suggests supervised PA is safe and feasible for children and adolescents during treatment—even during intensive treatment [29,30]—and that supervised PA may also reduce the severity of negative effects (eg, distress, fatigue, pain, nausea, physical and cognitive impairments [31,32]) and improve physical (eg, muscle strength and cardiorespiratory fitness); psychological (eg, emotional regulation and worry); social (eg, getting along with peers and building relationships); and cognitive (eg, reaction time and working memory) outcomes [30-37]. During treatment, PA may also support adaptive habit formation that carries forward into survivorship [38,39], a period in which PA continues to be associated with numerous health and quality of life benefits [40-44]. Finally, emerging evidence suggests PA may exert effects on inflammation [45-47], insulin sensitivity [48,49], and cardiovascular function [50,51]—mechanisms implicated in secondary cancers, morbidity, and premature mortality [52,53]. Observational evidence with young people who have completed treatment reinforces these findings, showing that PA is associated with a 39% relative reduction in secondary cancer risk [54] and is inversely related to morbidity and premature mortality [55].

Although evidence supporting the role of PA for children and adolescents diagnosed with cancer is compelling [56], it remains constrained by small sample sizes and varied, noncomparable measures, which hinder conclusive evidence and limit PA adoption. In response, guidelines based on extant evidence and expert consensus have been published. These guidelines suggest all children and adolescents diagnosed with cancer, both patients (on active treatment) and survivors (completed treatment), *move more* and offer recommendations for supporting PA, including supervision by trained exercise professionals [57,58]. Guidelines also underscore the pressing need for robust evidence on the effectiveness of PA while children and adolescents are on active treatment (patients). Despite the strong preliminary evidence and guideline statements, this cohort engages in significantly less PA than their healthy peers [59] and experience declines during treatment [60-62], which may persist following treatment [39]. Notably, supporting PA among pediatric patients with cancer may mitigate the side effects, promote a range of benefits, and foster PA adherence that lasts beyond treatment and into survivorship [63,64].

In Canada, pediatric cancer–specific PA opportunities (ie, PA interventions or programs developed for and delivered specifically to pediatric patients with cancer) are not currently part of standard care and exercise professionals (eg, kinesiologists, clinical exercise physiologists, and certified personal trainers) are not integrated into pediatric cancer care. Where interventions or programs do exist [65], they are located at single sites, often in research settings or foundations or organizations in major urban centers. Consequently, most patients have limited access to supervised PA during treatment, and few (if any) have access to trained exercise professionals. This lack of access, coupled with the substantial barriers patients and carers (eg, parents and guardians) face (eg, treatment-related effects, variable treatment timelines, periods of isolation and immunosuppression, and fears about safety [66,67]) can make engaging in PA challenging [66,67].

As a first step to addressing limited PA generally, and supervised PA specifically, during cancer treatment in Canada, we started in Alberta and considered local and site-specific capacity within the children’s hospitals as well as established PA programming [68] and developed IMPACT (IMplementation of Physical Activity for Children and adolescents on Treatment). IMPACT is a supervised PA intervention delivered by trained exercise professionals over videoconference [69]. Videoconference delivery was chosen to mitigate access barriers and make supervised PA available to more pediatric patients, regardless of geographic location, treatment protocols and timelines, and periods of isolation. Videoconference delivery omits the need for travel to in-person sessions, which can be difficult when experiencing challenging symptoms. This approach also has the potential to reduce demand on clinical sites (ie, pediatric oncology units) by enabling referral to supervised PA delivered “off-site” by a trained exercise professional. Relatedly, videoconference delivery allows exercise professionals to support patients remotely, thereby enhancing access to expertise that is otherwise unavailable within most pediatric cancer settings. Delivering PA by videoconference has been found to be feasible [70,71] and has been lauded as a promising PA delivery solution in pediatric cancer [72]—a small, geographically dispersed population in a large country such as Canada.

We are evaluating IMPACT in a type II hybrid effectiveness-implementation trial in Alberta. Recruitment closed at the end of December 2025. Clinician feedback and referral patterns indicate referral to supervised PA delivered “off-site” by a trained exercise professional is acceptable and feasible [73]. However, enrollment, trial retention, and adherence rates have been low, despite patients and carers initially expressing interest [74]. This pattern, particularly low enrollment, is commonly reported in the pediatric cancer and PA literature. Notably, for those who do enroll and complete the intervention, early visual analyses, an idiographic approach that involves inspecting individual participants’ data patterns over time to explore and describe effects, suggest IMPACT enhances quality of life, physical function, and PA levels. Taken together, findings signal a positive effect of IMPACT for those who participate, as well as a clear desire for PA support among patients, carers, and health care providers. Patterns also underscore that adaptations to implementation strategies are required. A deeper understanding of what patients and carers need and want from IMPACT is needed, including how consenting processes can be optimized. Collectively, the challenges encountered seemingly lie in the implementation of IMPACT.

Given the identified gaps; the demonstrated desire for PA from patients, carers, and health care providers; and funding support to scale IMPACT beyond Alberta, it is clear we must first reimagine IMPACT through active collaboration with research users—those who will refer to and/or use or benefit from IMPACT. This strategic co-adaptation will enhance the likelihood of relevance, acceptability, and uptake of IMPACT while also ensuring site-specific considerations are addressed across varied provincial contexts. Ultimately, this process will support bringing PA and thus enhanced health outcomes to more pediatric patients with cancer across Canada.

Over the next 5 years, our larger research program will (1) co-adapt IMPACT and prepare for scaling (phase 1) and (2) implement and evaluate co-adapted IMPACT across additional provinces in Canada (phase 2). The specific aims for phase 1 are detailed herein and include (1) identifying necessary IMPACT modifications, (2) examining site-specific factors influencing IMPACT implementation, and (3) developing an implementation research logic model for continued scaling.

## Methods

### Overview

An integrated knowledge translation and patient-oriented research approach and pragmatic orientation have been adopted [75-77]. A multiple-perspective study with key research user groups is underway. Relevant reporting guidelines will be followed when preparing and reporting results from this work (eg, FRAME [an expanded framework for reporting adaptations and modifications to evidence-based interventions] and Standards for Reporting Qualitative Research [78,79]). Selected implementation frameworks, tools, and models are informing this work, including the Consolidated Framework for Implementation Research (CFIR) 2.0 (a framework that identifies multilevel factors influencing implementation [80]), the CFIR-Expert Recommendations for Implementing Change Implementation Strategy Matching Tool (a matching tool that aligns implementation barriers with tailored strategies for effective change [81]), and the implementation research logic model (a model visualizing relationships between implementation determinants, strategies, mechanisms, and outcomes [82]).

### Ethical Considerations

Ethics approval was granted by the University of the Fraser Valley on June 10, 2025, through the BC Provincial Research Ethics Platform as a harmonized review (H25-00347). Recruitment commenced in July 2025 in British Columbia (BC), although exact dates were staggered and differed across hospital and organization sites pending time for relevant administrative reviews and approvals and amendments. Recruitment commenced in September 2025 in Ontario, although exact dates were staggered and varied pending time for administrative reviews and approvals. The Maritime provinces were onboarded on September 24, 2025, and ethics approval was granted by Island Health on March 11, 2026 (1032291). No research procedures occurred at sites prior to securing local approvals and no compensation is being provided to participants.

### Participants, Recruitment, and Informed Consent

Research user groups, including patients with and survivors of pediatric cancer or blood disorders, carers, health care providers and staff, and support organization personnel, are being recruited. Multiple approaches are being used to recruit members from each group.

#### Patients and Survivors

Both patients, on active treatment, and survivors, off or completed treatment, are being included in this phase of work, which differs from the IMPACT intervention and trial in Alberta. This decision was made based on the known PA benefits for patients and survivors and in consultation with clinical collaborators and support organization personnel partners to capture retrospective insights into perceptions of, and capacity to engage in, PA during treatment. Including survivors is also responsive to the known regional differences in PA opportunities and will inform whether an extension of the IMPACT intervention to survivors is warranted at sites across Canada.

In addition, both pediatric cancer and blood disorder diagnoses are included in this phase. This decision builds on the IMPACT intervention and trial delivery in Alberta wherein pediatric patients with blood disorders (ie, those diagnosed with any hematological condition) were eligible to participate. This cohort was included in Alberta due to pragmatic reasons (ie, they are treated on the same inpatient units; the intervention is delivered in a 1:1, supervised, tailored manner and is considered low risk; and emerging evidence suggests PA is safe and beneficial for children and adolescents diagnosed with blood disorders [83,84]). Consultations with clinical collaborators and support organization personnel partners supported the inclusion of children and adolescents diagnosed with blood disorders in this phase of work. As IMPACT is co-adapted for implementation and evaluation beyond Alberta, including perspectives from patients with and survivors of pediatric cancer and blood disorders and those who care for them will enable the determination of whether including both cancer and blood disorder diagnoses is indeed appropriate at sites across Canada or whether further site-specific modifications are required.

To be eligible, children and adolescents currently residing in BC, Ontario, or the Maritime provinces must (1) have been diagnosed with *any* cancer or blood disorder between 5 and 19 years of age, (2) be awaiting or receiving treatment or completed treatment within the past 5 or 10 years, (3) be less than 20 years of age at the time of study participation, and (4) be able to participate in English. Of note, with reference to eligibility criterion 2, in BC and Ontario, those who completed treatment in the past 5 years are being recruited; in the Maritime provinces, those who completed treatment in the past 10 years are eligible. This decision was pragmatic and based on the nature of patients and survivors seen by collaborators in the Maritime provinces. Patients and survivors are being recruited (1) through posters and advertisements at clinical sites, (2) through advertisements and emails sent by relevant support organizations, (3) by word of mouth (ie, snowball sampling), and (4) at selected sites with consent to contact forms being distributed by health care providers or staff.

#### Carers

To be eligible, carers residing in BC, Ontario, or the Maritime provinces must (1) self-identify as a carer for a child or adolescent diagnosed with any cancer or blood disorder between 5 and 19 years of age who is currently awaiting or receiving treatment or who completed treatment within the past 5 or 10 years in BC/Ontario and the Maritime provinces, respectively and (2) be able to participate in English. Carers will be recruited (1) through posters and advertisements at clinical sites, (2) through advertisements and emails sent by relevant support organizations, (3) by word of mouth (ie, snowball sampling), and (4) at selected sites with consent to contact forms being distributed by health care providers or staff.

#### Health Care Providers and Staff

To be eligible, health care providers and staff in BC, Ontario, or the Maritime provinces must be (1) currently working as a health care provider, allied health care provider, administrator, staff, or researcher; (2) responsible for care, coordination, and/or oversight in any capacity in the care of pediatric patients with or survivors of cancer and/or blood disorders; and (3) able to participate in English. Health care providers and staff are being recruited through (1) emails sent by the study staff to existing contacts and (2) word of mouth (ie, snowball sampling).

#### Support Organization Personnel

To be eligible, support organization personnel in BC, Ontario, or the Maritime provinces must be (1) employed (or recently previously employed) at a support organization that provides services to children and adolescents diagnosed with cancer or blood disorders and/or their carers; (2) responsible for care, coordination, and/or oversight in some capacity in the cancer and/or blood disorder support organization; and (3) able to participate in English. Support organization personnel are being recruited through (1) emails sent by the study staff to existing contacts and (2) word of mouth (ie, snowball sampling).

Potential participants are directed to contact the study staff via phone or email. Those who complete the consent to contact forms are followed up with by the study staff via the participant’s preferred method of communication (ie, email, phone call, or text message).

For all research user groups, study staff share study information and eligibility criteria, and if interested in participating, potential participants gain access to an electronic informed consent or assent form (as appropriate) hosted on SurveyMonkey. Of note, both child or adolescent assent and parental informed consent is completed for all patients and survivors, regardless of age. Carers, health care providers and staff, and support organization personnel complete informed consent only.

### Sample Size

Given the nature of this project, no formal sample size estimate was computed. Rather, targets for each participant group were set based on available data describing new diagnoses in BC, Ontario, and the Maritime provinces yearly [85], the estimated number of health care providers and staff at each site, and the number of known support organizations for pediatric patients with and survivors of cancer and blood disorders and survivors across sites. This information was supplemented with population considerations [86], study aims, guidance for information saturation [87], and existing conventions [88].

Approximately 6 to 12 pediatric patients with cancer and blood disorders and survivors including their carers, 6 to 12 health care providers and staff, and 1 to 2 support organization personnel per province (comprising multiple recruiting sites) are anticipated. These sample sizes are anticipated to be sufficient to ensure varied treatment status, wide age range, both sexes, and considerations for location (urban vs rural for patients, survivors, and carers).

### Data Collection

#### Descriptive Surveys

After electronic informed consent (and assent, as appropriate) is completed, participants automatically proceed to a brief descriptive survey. *Patients and survivors* (or their carers, if the minor is 5-13 years of age) are asked about their or their child’s age, biological sex, gender, diagnosis, medical treatments, current health status, and ethnicity or ancestral background. Additionally, PA levels are assessed using a modified Godin Leisure Time Exercise Questionnaire (mGLTEQ). The mGLTEQ asks participants to recall their (or their child’s) weekly PA over the past month and report average number of sessions (≥10 min in duration) per week and average duration or session spent in strenuous, moderate, mild, resistance, flexibility, and balance PA [89]. *Carers* are asked about their age, biological sex, gender, ethnicity or ancestral background, PA levels (via the mGLTEQ described earlier), marital status, education, income, and employment. *Health care providers and staff* and *support organization personnel* are asked about their age, biological sex, gender, title or role, years in the role, number of patients seen per week, educational background or training, and PA levels (using the mGLTEQ described earlier). All participant groups also answer questions to support scheduling or coordinating an interview at their convenience.

#### Interviews

Following survey completion, participants are contacted by the study staff to schedule their interview to explore necessary IMPACT modifications and site-specific factors influencing implementation. Interviews are scheduled at participants’ convenience and conducted via their preferred mode (ie, phone, videoconference, or in person, where the study staff are available). Participants may alternatively choose to respond to interview questions via email. Interviews with patient-carer or survivor-carer dyads may be conducted jointly or separately, depending on participant preference. Health care providers and staff and support organization personnel are interviewed individually. All interviews are conducted by the study staff with interview training, follow a semistructured interview guide, and are audio recorded and transcribed verbatim using Otter.ai.

The semistructured interview guide for each participant group was informed by the CFIR 2.0 [80], which contains 5 domains and 67 constructs. Domains include (1) innovation, (2) outer setting, (3) inner setting, (4) individuals, and (5) implementation processes. Interview guide questions explore aspects of each domain, site-specific adaptations necessary to enhance the relevance and acceptability of IMPACT, factors influencing the implementation of IMPACT across the system and individual levels, and strategies and processes to overcome barriers and maximize enablers (see Multimedia Appendix 1 for the interview guides). The interview guides were reviewed by carers and health care providers and were piloted among the study staff prior to use.

### Consultation

Concurrent to the data collection (ie, descriptive surveys and interviews) described earlier, 1:1 consultations are also being conducted with members of all research user groups in BC, Ontario, and the Maritime provinces who serve in a partner or collaborator role. These consultations provide an opportunity to obtain detailed feedback and engage in discussion on specific components of the IMPACT intervention and trial that are being raised in interviews (eg, intervention content, delivery processes, recruitment, and consenting procedures). Consultations are scheduled at partners’ and collaborators’ convenience and conducted via telephone or videoconference. Each consultation is tailored to the role and expertise of the partner or collaborator and is focused on specific topics or issues, enabling early feedback on data, offering suggestions, and supporting ongoing interpretation. Notes from consultations are documented and integrated with interview data to ensure that patient, survivor, carer, health care provider, and support organization personnel perspectives are informing the co-adaptation process. Finally, additional ancillary consultations are being conducted with Alberta-based carers of patients who participated in IMPACT, as well as clinical and support organization partners. These data are primarily being gathered to inform Alberta-based refinements and plans for sustainability; however, summaries will also be used to supplement data gathered herein and support the co-adaptation of IMPACT in BC, Ontario, and the Maritime provinces.

### Implementation Research Logic Model Development

#### Implementation Research Logic Model Draft

**Figure 1:**
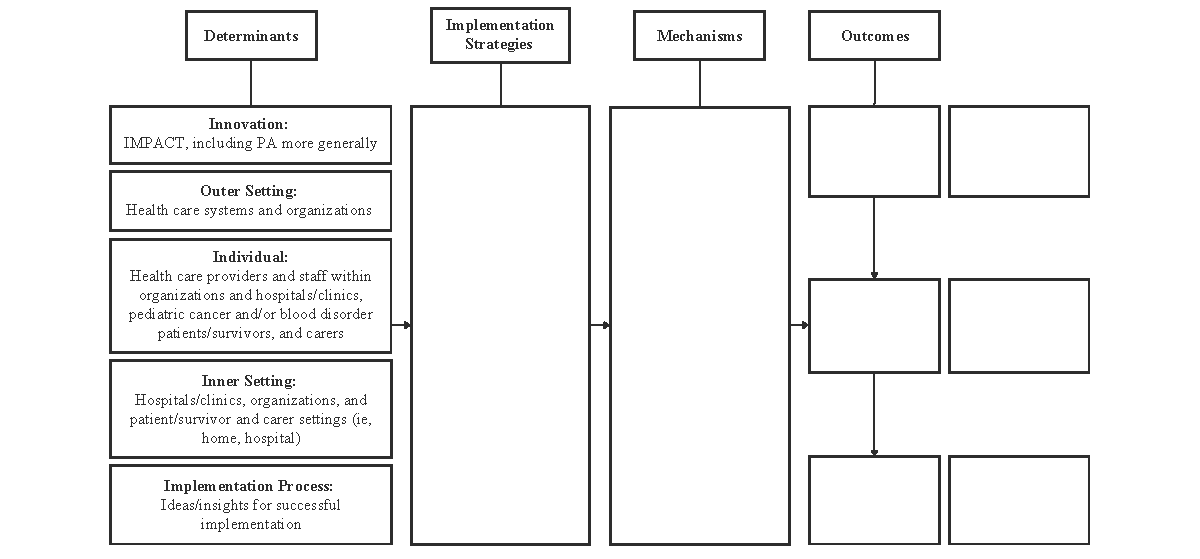
The Implementation Research Logic Model as described by Smith et al [82]. Determinants are factors that prevent or enable the implementation of IMPACT and a trial evaluating its effects (ie, the barriers and enablers). Implementation strategies are supports that are required to enhance the likelihood of successful uptake of IMPACT and its trial (eg, email reminders and educational presentations). Mechanisms are processes through which an implementation strategy operates to affect a desired outcome (eg, training). Outcomes are effects of deliberate action to implement IMPACT and its trial (may be indicators of implementation strategies or outcomes in relation to IMPACT and its trial; eg, effectiveness outcomes [eg, physical, psychosocial, or cognitive], implementation outcomes [eg, adherence, retention, or adverse events]). IMPACT: IMplementation of Physical Activity for Children and adolescents on Treatment; PA: physical activity.

Descriptive survey data to characterize the sample will be exported from SurveyMonkey and managed and analyzed in SPSS Statistics (IBM Corp) using descriptive statistics. Qualitative data will be managed and analyzed in NVivo 12 (Lumivero) and Microsoft Excel using framework analysis methods [90,91]. Qualitative data analysis will proceed in sequential steps, wherein the study staff will review transcripts to familiarize themselves with the data. A fully operationalized coding framework, developed by members of the research team, based on CFIR 2.0 [80] will be used to systematically code the data and identify determinants (barriers and enablers for implementation) and the strategies influencing IMPACT implementation (see Multimedia Appendix 2 for the fully operationalized coding framework) in each province. Where indicated, inductive coding will be applied to generate new or novel insights. Subsequently, inductively generated codes will be reviewed and grouped into internally homogeneous (ie, data within themes fit together meaningfully) and externally heterogeneous (ie, clear distinctions between each) themes. Following this, site-specific determinants (barriers and enablers) and implementation strategies will be documented. All codes across sites (and, where appropriate, themes) will be critically reviewed; discussed among the study team, partners, and collaborators; and mapped onto a draft implementation research logic model (Figure 1) [82].

#### Collaborative Refining of the Implementation Research Logic Model

Following mapping to the model, all participants will be recontacted and invited to review site-specific modifications and implementation considerations and provide feedback on the drafted model. This communication will take place by phone, videoconference, or email (as preferred by the participant). We expect that 1 to 3 iterations will take place (ie, that participants will be contacted to review iterations of the drafted models 1-3 times). This step is important, as it will enhance the likelihood of the resultant model reflecting study participants’, partners’, and collaborators’ perspectives. The results from this phase will include the analyzed interview dataset, a clear understanding of site-specific modifications and implementation strategies required, and a refined model showing pathways between implementation determinants, strategies, mechanisms, and outcomes.

### Research User Engagement

This research program and specific phase has adopted an integrated knowledge translation approach and has engaged research users as part of the study team from the outset. The involvement of Alberta-based patients, carers, health care providers, and support organization personnel will continue, and additional research users from BC, Ontario, and the Maritime provinces have been identified and involved. New research users were identified through professional and community conferences and workshops, networking with clinical collaborators and support organization partners, referral from existing collaborators, and snowball processes. Research users will continue to be identified as indicated via the same pathways. Current engagement mechanisms include advisory meetings and regular updates and newsletters. Planned engagement mechanisms include surveys and feedback loops and collaborative discussion sessions. Study team member composition includes patients or survivors and carers, health care providers, and support organization personnel, totaling over 35 research users as of January 2026 who are engaged in this work.

Beyond this, the project described herein seeks to capture and center research users’ perspectives via interviews and iterative data collating techniques. This supports developing, implementing, and evaluating PA interventions that are needed and wanted by pediatric patients with and survivors of cancer and blood disorders and those who care for and support them.

### Planned Knowledge Translation

We are publishing our process (including this protocol) and intend to share findings via a peer-reviewed publication. We will also share findings via site-specific presentations and will copresent (where possible) with research users at conferences and meetings. Aligned with our integrated knowledge translation approach, we have strong partnerships with research users and intend to collaboratively develop outputs (from peer-reviewed manuscripts to infographics to other dissemination tools) and copresent (ie, researcher and research user partnered presentations). We will deliver presentations (via videoconference and in person), create podcasts, draft media briefs, and leverage social media, and we have developed a website to share findings broadly.

## Results

This research program received 5 years of funding (awarded June 2023 and November 30, 2023), which was deferred and extended to start December 2024. The research program is therefore funded through until November 2029. Additional administrative and ethics approvals were secured across selected recruitment sites thereafter, although no research procedures occurred at each site until local approvals were obtained. Recruitment started in July 2025 and is commencing across sites in a staggered manner. Site-specific modification and implementation data are expected to be finalized in BC by March 2026, in Ontario by June 2026, and in the Maritime provinces by August 2026. Ethics submissions for a trial evaluating the co-adapted IMPACT are anticipated to be submitted in April 2026 for BC, in July 2026 for Ontario, and in September 2026 for the Maritime provinces. The full results (ie, all site-specific modifications and implementation strategies and the final version of the implementation research logic model) are expected to be submitted for publication in late 2026.

## Discussion

### Anticipated Findings

Pediatric cancer, although relatively rare, can have lasting physical, psychological, social, and cognitive effects. Despite the well-documented benefits of PA for this cohort [29-37], including published guidelines urging all children and adolescents diagnosed with cancer to move more [57], and the resultant development of IMPACT in Alberta, there are still several gaps that remain to be addressed. IMPACT, as it was implemented in Alberta, was limited by low enrollment, retention, and adherence rates, hindering the understanding of effectiveness and decisions about scaling and implementation. The proposed work will leverage lessons learned in Alberta and generate vital evidence to support PA evidence–based intervention co-adaptation and sustainable uptake from Alberta to BC, Ontario, and the Maritime provinces, varied regions with unique cancer care systems and PA support available.

Findings will represent a first step toward implementing and evaluating a reimagined IMPACT across Canada. The multisite database covering research user–identified factors influencing implementation across 3 Canadian provinces will directly inform supportive strategies. Outputs from this work will include site-specific implementation strategies, processes, and resources, as well as an implementation research logic model for ongoing scaling and implementation.

### Strengths

The proposed work has several strengths. First, adopting a collaborative approach centering research users’ perspectives will ensure a deeper understanding of real-world constraints. This will inform required modifications and increase the likelihood of identifying the breadth of barriers and facilitators that may support or hinder site-specific IMPACT implementation. Second, the pragmatic and broad enrollment criteria in this phase of work will allow for multiple and diverse perspectives to surface implementation challenges and inform potential strategies so that future implementation efforts are contextually relevant and feasible. This inclusive approach is already highlighting the needs and priorities of patients, survivors, carers, health care providers, and support organization personnel and is directly contributing to a reimagined version of IMPACT with greater potential to reach more young people affected by pediatric cancer. Third, the sample size estimates represent a notable strength and will result in a large dataset that will support exploration of varied experiences within and across provinces and enhance the transferability and credibility of findings. Fourth, using established implementation frameworks and models provides a systematic foundation for identifying barriers, facilitators, and strategies to guide scale-up.

### Limitations

Notwithstanding these strengths, there are also important considerations for the proposed work. First, all the study staff conducting interviews are trainees (at varying levels). This could influence the quality of data obtained or influence rapport building. To mitigate this, all study staff complete the same structured training that includes mock interviews and strategies for establishing rapport and effective probing and have their initial interviews audited to identify areas for improvement. Second, as an early-career investigator, the project lead (first author) is actively developing relationships and seeking to build a rich network comprising varying organizations that serve children and adolescents diagnosed with cancer or blood disorders and their carers. Finally, the study team (including research assistants) are positioned outside of hospital settings, which may present challenges for outreach and recruitment. To address this, the study team are maintaining active communication with health care providers and staff and support organization personnel involved in recruitment. Uniquely, this consideration and positioning also offers a valuable opportunity to establish clear referral pathways and processes that connect families to PA, including the reimagined IMPACT, extending access beyond traditional clinical environments—a key component of this research program.

### Conclusions

Overall, this phase, within the broader research program described, represents an important and timely step toward ensuring that more pediatric patients with and survivors of cancer and blood disorders have access to supervised PA. By working together with patients, survivors, carers, health care providers and staff and support organization personnel to understand modifications and site-specific considerations, we will generate practical and transferable insights to guide an implementation research logic model and subsequent implementation and evaluation of the reimagined IMPACT across additional provinces. This work will enhance the likelihood of broader applicability and uptake across varied Canadian contexts.

## Appendix

Multimedia Appendix 1 Interview guides.

Multimedia Appendix 2 Fully operationalized coding framework.

## Abbreviations

CFIR: Consolidated Framework for Implementation Research
FRAME: an expanded framework for reporting adaptations and modifications to evidence-based interventions
IMPACT: IMplementation of Physical Activity for Children and adolescents on Treatment
PA: physical activity

## Data Availability

Data sharing is not applicable to this paper as no datasets were generated or analyzed.
