# Supplementary material for: Moving Forward Together: A Protocol to Co-Adapt and Scale a Videoconference-Delivered Physical Activity Intervention for Children and Adolescents Diagnosed With Cancer or Blood Disorders in British Columbia, Ontario, and the Maritime Provinces
Source: JMIR Res Protoc. 2026 May 29;15:e92574. doi: 10.2196/92574 (PMC13263662; doi:10.2196/92574)
Supplement: Multimedia Appendix 1 [file resprot_v15i1e92574_app1.docx]

**Supplementary File 1. Interview Guides**

**Children and Adolescents Diagnosed with Cancer Semi-Structured Interview Guide**

CHILDREN (5-10+)

[START AUDIO RECORDER]

Hello, my name is [INSERT NAME], and I am [INSERT ROLE WITH STUDY], it is nice to meet you. Today we are going to talk about physical activity. That means moving your body and using energy. It includes fun things like running, jumping, playing tag, riding a bike, dancing, or even helping carry things. If you’re not sitting still, and you’re moving around, you’re being active.

I am going to ask you some questions about physical activity and then you can tell me what you think, like, and dislike because we want you to help us make a physical activity program for kids like you! If you do not want to answer any of these questions, you don’t have to. You can skip any questions you want.

Do you have any questions before we begin?

[If yes, answer accordingly].

[If not, continue with questions below].

1. Do you like to move/play?

[If yes] What do you like to do? Is this the same/different than before your cancer/blood disorder treatment?

[If not] Why not? What else do you like to do (e.g., arts & crafts, read)? Is this the same/different than before your cancer/blood disorder treatment?

Since the reason we are chatting today is to get your help to create a physical activity program for kids like you, I am going to tell you a bit about a program we made in Alberta. I’d like you to tell me what you think and what you would want us to change or do in *this* program.

**Innovation**

1. In the physical activity program we made in Alberta, we are offering physical activity during treatment. What do you think about this? When do you think we should offer this program you help create? During treatment for cancer or blood disorder? After treatment? Or both?

PROBE: What do you think about physical activity for kids with cancer or blood disorder?

PROBE: Have you done a physical activity program before? Why/Why not?

PROBE: Would you like to do a physical activity program [during/after] treatment [depending on the stage they are currently; eg, if on-treatment ask if they’d want to do a physical activity program during treatment]? Why/Why not?PROBE: Do you think the other kids you know with cancer or blood disorder would want to do a physical activity program?

**Implementation Process/Inner Setting**

1. The kids who are doing the physical activity program in Alberta learn about the program through posters at the hospital, their parents, or their doctor.

PROBE: How would you like to learn about a physical activity program? Would you like to hear from a member of the research team in person (i.e. at the hospital/clinic?)

PROBE: What would be the best way to get you interested in this program?

PROBE: Is there anyone else who should tell you about it?

PROBE: In Alberta, over 95 kids have heard about the program, but only 15 have participated. Do you have any ideas for how we could share this program to other kids? How do you think we can involve more kids to participate?

**Innovation**

1. In the physical activity program in Alberta, kids meet someone like me on a videocall 2-3 times/week, for 3 months (12 weeks) and move together. Sometimes this looks like playing games, sometimes this looks like stretching and breathing, and sometimes we use more energy to do things like lunging and squatting. Sometimes kids are at their home and sometimes they are at the hospital or other places when we meet to do physical activity. What do you think about this?

PROBE: Would you want to do physical activity on a videocall? Why? Why not?

PROBE: How would you like to do physical activity? Videocall? In-person? Other?

PROBE: Would you want to do physical activity 2-3 times/week? Why?/Why not? How

long would you want each session to be?

PROBE: Would you want to do it for 3 months? Why? Why not?

PROBE: How many times/week would you want to do physical activity for? And how long?

**Innovation**

1. In the physical activity program in Alberta, we try to make the physical activity we do as fun as possible! We do things like play games based on the kids’ favourite movie or book characters, or their favourite sports. So, we might play hide and seek, or we might take our favourite animal for a little walking tour. Sometimes, if kids want, their sibling or parent might join in.

PROBE: What do you think about this?

PROBE: Do you think you’d like it? Why? Why not?

PROBE: How could we make physical activity more fun for you?

PROBE: What type of movement would you like to do?

PROBE: Would you want anyone (parent, friend, other kids with cancer/blood disorder) to be doing the program with you?

**Innovation**

1. In the physical activity program in Alberta, we ask parents to be present during the program for safety.

PROBE: What do you think about this?

**Outer/Inner Setting**

1. In the program in Alberta that is offered by videocall 2-3 times/week over 3 months, what sounds hardest for you to do? What seems hard about it? How can we make it easier?

PROBE: What things make (or could make) physical activity hard for you right now?

**Outer/Inner Setting**

1. In the program in Alberta that is offered by videocall 2-3 times/week over 3 months, what sounds easiest about it? What should we continue to do to make it easy?

PROBE: What things make (or could make) physical activity easier for you?

**Innovation/Individuals/Outer Setting**

1. In the physical activity program that I have been describing in Alberta, we ask the kids lots of questions before and after the program. This is so we can see how they feel when they do it. We also measure things like how strong they are.

PROBE: What types of things would you want us to measure? This could be things that are important to you, like feeling better/happier.

PROBE: What would you want us to ask you about before/after a physical activity program?

PROBE: Is there anything you think we, as researchers, should know more about physical activity and kids with cancer or blood disorders?

**Innovation/Individuals**

1. In the physical activity program in Alberta, we give kids a Fitbit that they can wear throughout the program. There are a few times where we ask them to wear the Fitbit for a week, and we track how many steps they take.

PROBE: Do you know what a Fitbit is? Have you used one before?

PROBE: What do you think about using a Fitbit?

PROBE: Would you prefer something else?

PROBE: What would you want a device to look like or tell you?

**Innovation/Individuals**

1. In the physical activity program in Alberta, we also give kids a yoga mat that they can use during the program and keep it once they are done.

PROBE: What do you think about this?

PROBE: Is there anything else you think you might need to participate in a physical activity program?

PROBE: Is there anything we can give you that would increase your motivation to do physical activity?

PROBE: I should also share that with the funding we have; we are able to provide devices (eg, iPads) with data so that those without access to an iPad or internet can still take part. What do you think of this?

**Other**

1. Is there anything else you would like to share with us?

**Children and Adolescents Diagnosed with Cancer Semi-Structured Interview Guide**

[TEENAGERS/ADOLESCENTS (11+)]:

[START AUDIO RECORDER]

Hello, my name is [INSERT NAME], and I am [INSERT ROLE WITH STUDY], it is nice to meet you. Today we are going to talk about physical activity. When I say physical activity, I mean any movement your body makes that uses energy. This includes things like walking, running, playing sports, dancing, or even doing chores like cleaning or carrying groceries. It can be light, like stretching, or more intense like sprinting or lifting.

I am going to ask you some questions about physical activity and then you can tell me what you think, like, and dislike because we want you to help us make a physical activity program for kids like you! If you do not want to answer any of these questions, you don’t have to. You can skip any questions you want.

Since we are adapting in BC, ON, and the Maritime provinces we want to make sure that we are adapting the program to your location. What hospital or where did you hear about us?

Do you have any questions before we begin?

[If yes, answer accordingly].

[If not, continue with questions below].

1. Do you like physical activity?

[If yes] What physical activity do you like? Is this the same/different than before your treatment?

[If not] Why not? What else do you like to do (e.g., arts & crafts, read)? Is this the same/different than before your treatment?

Since the reason we are chatting today is to get your help to create a physical activity program for kids like you, I am going to tell you a bit about a program we made in Alberta. I’d like you to tell me what you think and what you would want in this program.

I am hoping you saw the document that was sent when this interview was scheduled. The document provides a brief overview of IMPACT and how it’s being implemented and evaluated in Alberta.

Briefly, IMPACT is a physical activity program for kids with any cancer *or* blood disorder, who are between the ages of 5-18, who are awaiting-, on-, or within 3 months of completing treatment. The physical activity program is delivered by videocall using Zoom, 1 to 1 by a trained exercise professional for 3 months (12 weeks). The sessions are 15-45 minutes and offered 2-3 times/week. As well, each session is tailored based on how the patient is feeling (energy, fatigue, treatment-related side-effects), their age, and their preferences.

We are interested in testing how well this program works for kids like you – before the program, right after the program and later on.

Do you have any questions about this?

**Innovation**

1. In the physical activity program we made in Alberta, we are offering physical activity during treatment. What do you think about this? When do you think we should offer this program you help create? During treatment for cancer or blood disorder? After treatment? Or both?

PROBE: What do you think about physical activity for kids affected by cancer and blood disorders?

PROBE: Have you done a physical activity program before? Why/Why not?

PROBE: Would you like to do a physical activity program [during/after] treatment [depending on the stage they are currently; eg, if on-treatment ask if they’d want to do a physical activity program during treatment]? Why/Why not?

PROBE: Do you think the other kids you know with cancer or blood disorder would want to do a physical activity program?

**Implementation Process/Inner Setting**

1. The kids who are doing the physical activity program in Alberta learn about the program through posters at the hospital, their parents, or their doctor.

PROBE: How would you like to learn about a physical activity program? Would you like to hear from a member of the research team in person (i.e. at the hospital/clinic?)

PROBE: What would be the best way to get you interested in this program?

PROBE: Is there anyone else who should tell you about it?

PROBE: In Alberta, over 95 kids have heard about the program, but only 15 have participated. Do you have any ideas for how we could share this program to other kids? How do you think we can involve more kids to participate?

**Innovation**

1. In the physical activity program in Alberta, kids meet someone like me on a videocall 2-3 times/week, for 3 months (12 weeks) and move together. Sometimes this looks like playing games, or stretching and breathing, or we do things like lunging and squatting. Sometimes kids are at their home and sometimes they are at the hospital or other places when we meet to do physical activity.

PROBE: What do you think about this?

PROBE: Would you want to do physical activity on a videocall? Why? Why not?

PROBE: How would you like to do physical activity? Videocall? In-person? Other?

PROBE: Would you want to do physical activity 2-3 times/week? Why? Why not? How long would you want each session to be?

PROBE: Would you want to do it for 3 months? Why? Why not?

PROBE: How many times/week would you want to do physical activity for? And how long?

**Innovation**

1. In the physical activity program in Alberta, we try to make the physical activity we do as fun as possible! We do things like play games based on the kids’ favourite movie or book characters, or their favourite sports. So, we might do more structured exercises like circuits – from either seated or standing. Sometimes, if kids want, their sibling or parent might join in.

PROBE: What do you think about this?

PROBE: Do you think you’d like it? Why? Why not?

PROBE: How could we make physical activity more fun for you?

PROBE: What type of movement would you like to do?

PROBE: Would you want anyone (parent, friend, other kids with cancer/blood disorder) to be doing the program with you?

**Innovation**

1. In the physical activity program in Alberta, we ask parents to be present during the program for safety.

PROBE: What do you think about this?

**Outer/Inner Setting**

1. In the program in Alberta that is offered by videocall 2-3 times/week over 3 months, what sounds hardest for you to do? What seems hard about it? How can we make it easier?

PROBE: What things make (or could make) physical activity hard for you right now?

**Outer/Inner Setting**

1. In the program in Alberta that is offered by videocall 2-3 times/week over 3 months, what sounds easiest about it? What should we continue to do to make it easy?

PROBE: What things make (or could make) physical activity easier for you?

**Innovation/Individuals/Outer Setting**

1. In the physical activity program that I have been describing in Alberta, we ask the kids lots of questions before and after the program. This is so we can see how they feel when they do it. We also measure things like how strong they are.

PROBE: What types of things would you want us to measure? This could be things that are important to you, like feeling better/happier.

PROBE: What would you want us to ask you about before/after a physical activity program?

PROBE: Is there anything you think we, as researchers, should know more about physical activity and kids with cancer or blood disorders?

**Innovation/Individuals**

1. In the physical activity program in Alberta, we give kids a Fitbit that they can wear throughout the program. There are a few times where we ask them to wear the Fitbit for a week, and we track how many steps they take.

PROBE: Do you know what a Fitbit is? Have you used one before?

PROBE: What do you think about using a Fitbit?

PROBE: Would you prefer something else?

PROBE: What would you want a device to look like or tell you?

**Innovation/Individuals**

1. In the physical activity program in Alberta, we also give kids a yoga mat that they can use during the program and keep it once they are done.

PROBE: What do you think about this?

PROBE: If we were to provide patients with something to support their participation in the program as I have described it, what do you think it would be?

PROBE: Is there anything else you think you might need to participate in a physical activity program?

PROBE: Is there anything we can give you that would increase your motivation to do physical activity?

PROBE: I should also share that with the funding we have; we are able to provide devices (eg, iPads) with data so that those without access to an iPad or internet can still take part. What do you think of this? How do you think we should advertise this? Do you think there are other things we should include/offer to ensure as many kids can benefit as possible?

**Other**

1. Is there anything else you would like to share with us?

**Caregivers of Children and Adolescents Diagnosed with Cancer Semi-Structured Interview Guide**

[PARENTS/CAREGIVERS]

[START AUDIO RECORDER]

Hello, my name is [INSERT NAME], and I am [INSERT ROLE WITH STUDY], it is nice to meet you. Today we are going to talk about physical activity for kids affected by cancer and/or a blood disorder. When I say physical activity, I mean any movement your body makes that uses energy. This includes things like walking, running, playing sports, dancing, or even doing chores like cleaning or carrying groceries. It can be light, like stretching, or more intense like sprinting or lifting.

As you may recall from the informed consent that you completed, we are conducting this study to adapt of an existing physical activity program, called IMPACT, that we are studying in Alberta.

Since we are adapting in BC, ON, and the Maritime provinces we want to ensure we are tailoring the program to your location. What hospital or where did you hear about us?

We have funding to bring this program to [INSERT LOCATION], and as a first step, to we want to speak with caregivers like you and as well as healthcare providers, personnel at support organizations, and patients to understand if or how we need to adapt IMPACT and what we need to consider to successfully run a study evaluating IMPACT here.

Essentially, the goal is for us to make sure we are considering barriers so that we can develop strategies to overcome them in advance and to identify facilitators, so we can do what we can to maximize them.

Did you have any questions for me after you read the consent form? Or question about this research overall?

[If yes, answer accordingly].

[If not, continue with questions below].

1. Does your child like physical activity?

[If yes] What physical activity do they like? Is this the same/different than before their treatment?

[If not] Why not? What else do they like to do (e.g., arts & crafts, read)? Is this the same/different than before their treatment?

The reason we are chatting today is to get your help to create a physical activity program for kids affected by cancer and/or blood disorder. So, I am going to tell you a bit about it and then I’d like you to tell me what you think.

I am hoping you saw the document that was sent when this interview was scheduled. The document provides a brief overview of IMPACT and how it’s being implemented and evaluated in Alberta.

Briefly, IMPACT is a physical activity program for kids with any cancer *or* blood disorder, who are between the ages of 5-18, who are awaiting-, on-, or within 3 months of completing treatment. The physical activity program is delivered by videoconference using Zoom, 1:1 by a trained exercise professional for 12 weeks. The sessions are 15-45 minutes and offered 3 times/week over 12 weeks. As well, each session is tailored based on how the patient is feeling (energy, fatigue, treatment-related side-effects), their age, and their preferences.

Do you have any questions about this?

We are interested in testing how well this program works for kids like your child – before the program, right after the program and later on.

We are evaluating this program using a study design that allows us to look at things that influence implementing the program and effectiveness – so we are measuring a range of outcomes before and after the program and at 2 follow-up times to see if there’s any change. I should note that our study design also enables *all* kids with a diagnosis of cancer or blood disorder to take part – there is no randomization, and this is because we know that physical activity is safe, feasible, beneficial, and recommended for pediatric cancer patients.

Do you have any questions?

**Innovation**

1. The physical activity program in Alberta is offered to children and teens (5-18) who are on-treatment for cancer or blood disorder. What do you think about this? When should we offer this program? During treatment for cancer or blood disorder? After treatment? Or Both?

PROBE: What is your sense/thoughts/feelings about this?

PROBE: What do you think about physical activity for kids affected by cancer and blood disorders? For your child?

PROBE: Has your child done a physical activity program before? Why/Why not?

PROBE: Do you think they would want to do a physical activity program during/after treatment? Why/Why not? Would you want them to do a physical activity program during/after treatment?

PROBE: Do you think other kids with cancer or blood disorder (and their caregivers) would be interested in something like this?

PROBE: What do you think about including kids both affected by cancer and blood disorder?

PROBE: What are your thoughts on the age range of 5-18 years? Do you believe this age range should be increased to 19-20 year olds?

**Implementation Process**

1. The kids who are doing the physical activity program in Alberta learn about the program through posters at the hospital, their parents, or their doctor. Specifically, at hospitals, caregivers complete a consent to contact form, do you know what this is? [if not, “this form is completed by caregivers where they indicate if they are interested in receiving more information about the study and their preferred method of contact (i.e., phone call, text, email) which is then sent to the study staff who then take over communication]. Other recruitment methods include advertising (e.g., social media, emails from support organizations), and word of mouth (i.e., past participant referral).

PROBE: What is your sense/thoughts/feelings about this?PROBE: How would you like to learn about a physical activity program for your child? Would you like to hear from a member of the research team in person (i.e. at the hospital/clinic?)

PROBE: What about the consent to contact process seems easy or hard for you?

PROBE: What other way would you like to be contacted? Do you know other potential ways we could use to reach families so that they are aware of this program?

PROBE: What would be the best way to get you/your child interested in this program?PROBE: Is there anyone else who should tell you or your child about it?

1. To date, we have had over 95 kids referred, but only 15 have consented, enrolled, and participated.

PROBE: People seem interested but then do not enroll, do you have any ideas for why kids may not be participating? Do you have any ideas for how we could close that gap and ensure more kids who are referred, consent and enrol?

PROBE: What do you think we can do to attract more kids, and their caregivers, to participate? For this program we are creating, how do you think we can involve more kids?

PROBE: What would attract you/your child to participate?

**Innovation**

1. In the physical activity program in Alberta, kids meet someone like me on a videocall 2-3/week, for 3 months (12 weeks) and move together. Sometimes this looks like playing games, or stretching and breathing, or we do things like lunging and squatting.

PROBE: What is your sense/thoughts/feelings about this?

PROBE: Do you think your child would benefit from a program like this? Why/Why not?

PROBE: Do you think your child would want to do physical activity on a videocall?

PROBE: Would you want your child to do it for 3 months? Why? Why not?

PROBE: How many times/week do you think your child would want to do physical activity for? And how long each session? And how long overall [ie, duration; for how many weeks/months]? And what about you? How many times/week would you want your child to do physical activity for? And how long each session? And how long overall [ie, duration; for how many weeks/months]?

**Innovation**

1. In the physical activity program in Alberta, we try to make the physical activity we do as fun as possible! We do things like play games based on the kids’ favourite movie or book characters, or favourite sports. So, we might play hide and seek, or we might take our favourite animal for a little walking tour for your kids or do a more structured workout for teens. Sessions can be done seated or standing depending on how a kid is feeling.

PROBE: What is your sense/thoughts/feelings about this?

PROBE: Do you think your child would like something like this? Why? Why not?

PROBE: How could we make it more fun for your child?

PROBE: What type of movement would your child like to do? What would you like them to do in a program like this?

PROBE: Do you have any other suggestions for activities to include in these sessions?

**Implementation Process**

1. The physical activity program that is offered in Alberta was developed based on input from semi-structured interviews like this with healthcare providers and staff from the two children’s hospitals in Alberta and international experts (researchers, qualified exercise professionals) who deliver physical activity to kids affected by cancer. We now have a patient advisory board comprised of patients, caregivers, healthcare providers, community partners, and the study staff, who review and comment on aspects of IMPACT and the trial.

PROBE: What are your thoughts and feelings about this development approach?

PROBE: Should we consider anything/anyone else?

PROBE: When we are adapting, is there a perspective we might be missing?

**Innovation**

1. One way we ensure safety is to first have healthcare providers and allied providers, like physiotherapists and nurses provide medical clearance for patients who will participate.

PROBE: What is your sense/thoughts/feelings about this?

PROBE: Would you have any challenges with obtaining medical clearance?

**Innovation**

1. Another consideration for safety is that we ask a caregiver or adult to be present during the sessions. This could mean being in the same room, doing the activities, or another room in the house.

PROBE: What is your sense/thoughts/feelings about this? What would be your preference?

PROBE: Do you think this would be easy or difficult for you or other caregivers based on your experience?

PROBE: Is there anything else we should consider re: safety?

**Innovation**

1. The physical activity program is delivered by a trained qualified exercise professional who has experience delivering online physical activity for individuals (children, adolescents, young adults, adults) affected by cancer and have undergone study-specific training.

PROBE: What is your sense/thoughts/feelings about this?

PROBE: Would you feel comfortable having your child participate given who is delivering it?

PROBE: Is there anything else we should consider?

**Outer/Inner Setting**

1. In the program in Alberta that is offered by videocall 2-3 times/week over 3 months, might make it harder for your child to do? What seems hard about it? How can we make it easier?

PROBE: What things make (or could make) supporting your child to do physical activity hard for you right now? What resources might you need?

**Outer/Inner Setting**

1. In the program in Alberta that is offered by videocall 2-3 times/week over 3 months, what sounds easiest about it for your child to do? What should we continue to do to make it easy?

PROBE: What things make (or could make) supporting your child to do physical activity easier for you right now? What resources might you need?

**Innovation/Individuals**

1. In the physical activity program I have been describing in Alberta, we ask the kids and parents lots of questions before and after the program so we can see how they feel when they do it. We also measure things like how strong they are. I can give you examples of what we are currently measuring in Alberta if you’d like? [If yes, list: the current IMPACT trial is exploring patient’s objective PA (primary effectiveness outcome) and secondary effectiveness outcomes of patient- and caregiver-reported outcomes (i.e., patient and caregiver-reported PA and patient’s quality of life, symptoms, cognitive function, resource use), and physical fitness outcomes (i.e., patient’s aerobic endurance, lower body flexibility, shoulder flexion range of motion, balance, functional mobility)].

PROBE: What outcomes are important to you and your child? What do you think we should measure?

PROBE: If we could only measure 3 things, what would you suggest? Why?

PROBE: What would you want us to ask you or measure before/after physical activity?

PROBE: Is there anything you think we, as researchers, should know more about kids like yours?

PROBE: What would make it easier or harder for you and your child to complete these questions.

**Innovation/Individuals**

1. In the physical activity program in Alberta, participants are given a Fitbit to use throughout the study. At the same timepoints as the other assessments, baseline (week 0), post-program (week 12) and at follow-up timepoints (week 24, week 52), we ask participants to wear the Fitbit for one week and complete a tracking log to indicate how long they wore the Fitbit. We are assessing the number of steps they take at these various timepoints.

PROBE: Do you know what Fitbits are? Have you ever worn one? Has your child?

PROBE: Would your child be able to wear a Fitbit at these different timepoints? If no, what can we do to support your child to wear this?

PROBE: What do you think about kids wearing these at different timepoints?

PROBE: Would you or your child would prefer something else?

PROBE: What would you or your child want the device to look like or tell you/them?

**Innovation/Individuals**

1. In Alberta, participants are also given a yoga mat that they can use during the program and are able to keep once they are done in the program.

PROBE: What is your sense/thoughts/feelings about this?

PROBE: Is there anything you think your child would need to participate in a physical activity program? Or that would be useful/motivating if they were able to keep?

PROBE: Is there anything we can give you that would increase your motivation to do physical activity?

PROBE: Is there anything we should consider?

PROBE: I should also share that with the funding we have; we are able to provide devices (eg, iPads) with data so that those without access to an iPad or internet can still take part. What do you think of this? How do you think we should advertise this? Do you think there are other things we should include/offer to ensure as many kids can benefit as possible?

**Other**

1. Is there anything else you would like to share with us?

**Healthcare Provider and Staff Semi-Structured Interview Guide**

[START AUDIO RECORDER]

Hello, my name is [INSERT NAME], and I am [INSERT ROLE WITH STUDY], it is nice to meet you. As you may recall from the informed consent that you completed, we are conducting this study to adapt an existing physical activity program, called IMPACT to [INSERT LOCATION].

Before we begin, we just want to confirm your location. We are adapting in BC, ON, and the Maritime provinces and want to ensure we are tailoring the program to your location. What city are you currently located in and what hospital do you work at?

As a first step, we want to speak with you, and people in similar roles as you, as well as personnel at support organizations and patients and their caregivers to understand if or how we need to adapt IMPACT and what we need to consider to successfully run a study evaluating IMPACT at your site.

Essentially, the goal is for us to make sure we are considering barriers so that we can develop strategies to overcome them in advance and to identify facilitators, so we can do what we can to maximize them.

Did you have any questions for me after you read the consent form? Or question about this research overall?

[If yes, answer accordingly].

[If not, continue with questions below].

I am hoping you saw the document that was sent when this interview was scheduled. The document provides a brief overview of IMPACT and how it’s being implemented and evaluated in Alberta.

Briefly, IMPACT is a physical activity program for children and adolescents with any **cancer or blood disorder**, who are **between the ages of 5-18**, who are **awaiting-, on-, or within 3 months of completing treatment**. The physical activity program is delivered by **videoconference** using Zoom, 1:1 by a trained exercise professional **2-3 times/week over 3 months (12 weeks).** Each session is 15-45 minutes long and is tailored based on how the patient is feeling (energy, fatigue, treatment-related side-effects), their age, and their preferences.

Do you have any questions about this?

We are evaluating this program using a study design that allows us to look at things that influence implementing the program and effectiveness – so we are measuring a range of outcomes before and after the program and at 2 follow-up times to see if there’s any change. I should note that our study design also enables *all* patients to take part – there is no randomization, and this is because we know that physical activity is safe, feasible, beneficial, and recommended for pediatric cancer patients.

Do you have any questions?

Overall, what modifications or tailoring do you believe the program needs to undergo to fit within your setting? What challenges/complications associated with offering a physical activity program in [INSERT LOCATION] do you foresee?

I will now ask you some specific questions about elements of the physical activity program in Alberta and your thoughts as to what needs to change/be considered to work in your setting/community.

**Innovation**

1. So, as we move through the question I have for you, I will first tell you what we are currently doing in Alberta and then ask for your thoughts. We will start with participants.

First, you should know that IMPACT and the trial were developed using the literature and resources we have available in pediatric cancer. However, given pragmatic nature of the program, early findings for safety of 1:1, tailored physical activity, and similarities in some treatment and thus side-effects, we decided to offer the program to children and adolescents affected by blood disorder as well. As mentioned, children and adolescents diagnosed with any cancer *or* blood disorders, who are between the ages of 5-18, who are awaiting-, on-, or within 3 months of completing treatment are eligible.

PROBE: What is your sense/thoughts/feelings about this?

PROBE: What do you think about physical activity for pediatric cancer and blood disorder patients and survivors?

PROBE: What are your thoughts on including both pediatric cancer and blood disorder patients in this program? Are there any considerations that need to be taken in? Why/Why not?

PROBE: What are your thoughts on the age range of 5-18 years? Do you believe this age range should be increased to 19-20 year olds?

PROBE: What about only including children and adolescents on-treatment?

PROBE: Is there a need to include children and adolescents' post-treatment? Why or why not?

**Implementation Process/Inner Setting**

1. In our physical activity program in Alberta, we are recruiting patients through consent to contact forms, do you know what this is? [if not, “this form is completed by caregivers where they indicate if they are interested in receiving more information about the study and their preferred method of contact (i.e., phone call, text, email) which is then sent to the study staff who then take over communication]. Other recruitment methods include advertising (e.g., social media, emails from support organizations), and word of mouth (i.e., past participant referral).

PROBE: What is your sense/thoughts/feelings about this this approach?

PROBE: Specifically, what do you think of consent to contact? Do you think anyone on your team (e.g., support staff) has capacity to send these? Would someone picking them up regularly be better?

PROBE: Do you think your site/team would be willing to have a research staff member on site to facilitate recruitment? Has this been done before at your site? Was it successful? What might we need to consider with this approach?

PROBE: What is the best way (if we do consent to contact) to do this at your site with the least amount of burden on people like you and your team?

PROBE: In terms of posters would you hang these up at your site and refer your patients to these?

PROBE: What has worked well in the past to support recruitment to trials – including physical activity trials?

PROBE: What can we do to make it easier?

PROBE: What additional resources/documents might be needed?

PROBE: How else should families hear about the program?

1. In Alberta, over 95 children and adolescents have heard about the program, but only 15 have anticipated. Have you seen this issue before with studies at/recruiting through your site?

PROBE: People are interested but then do not enroll, do you have any ideas for how we could close that gap and ensure more children and adolescents who are referred, consent and enrol?

PROBE: Do you have recruitment methods that have proven to be successful?

PROBE: What do you think we can do to attract more children, adolescents, and their caregivers, to participate? For this program we are creating, how do you think we can involve more children and adolescents?

**Implementation Process**

1. The physical activity program that is offered in Alberta was developed based on input from semi-structured interviews like this with healthcare providers and staff from the two children’s hospitals in Alberta and international experts (researchers, qualified exercise professionals) who deliver physical activity to pediatric cancer and blood disorder patients and survivors. We now have a patient advisory board comprised of patients, caregivers, healthcare providers, community partners, and the study staff, who review and comment on aspects of IMPACT and the trial.

PROBE: What is your sense/thoughts/feelings about this development approach?

PROBE: Should we consider anything/anyone else?

PROBE: When we are adapting, is there a perspective we might be missing?

**Implementation Process/Innovation/Inner Setting**

1. In the physical activity program that is offered in Alberta, one way we are ensuring safety is to first have healthcare providers and allied providers, like physiotherapists and nurses provide medical clearance for the patients who will participate.

PROBE: What is your sense/thoughts/feelings about this?

PROBE: Do you think it makes sense that any healthcare or allied provider can provide clearance?

PROBE: Do you envision any challenges with providing the medical clearance?

PROBE Have you had to do this in the past for other studies? What worked or didn’t work?

PROBE: What would be the easiest way for us to facilitate obtaining medical clearance? What pathway for information sharing would you propose? How could we make it easier for your site?

PROBE: Is there anything else we should consider?

**Implementation Process/Innovation/Inner Setting**

1. In the physical activity program that is offered in Alberta, another consideration for safety is that we ask a caregiver or adult to be present during the sessions. This could mean being in the same room, doing the activities, or another room in the house.

PROBE: What is your sense/thoughts/feelings about this?

PROBE: Is there anything else we should consider re: safety?

**Implementation Process/Innovation**

1. In the physical activity program that is offered in Alberta, the physical activity program is delivered by a trained qualified exercise professional who has experience delivering online physical activity for individuals (children, adolescents, young adults, adults) affected by cancer and have undergone study-specific training.

PROBE: What is your sense/thoughts/feelings about this?

PROBE: Would you feel comfortable promoting this study given who is delivering it?

PROBE: Is there anything else we should consider?

**Inner Setting**

1. Do you think healthcare providers and staff at your site would be interested and/or supportive of a physical activity program like this?

PROBE: Are there people who might be opposed or unsupportive? What might we do to leverage their support? How could we gain their support?

PROBE: What kinds of changes might be needed to facilitate referrals/recruitment to this program at your site? (E.g., structural, work, IT)

PROBE: What changes need to be made to embed referral to the physical program into your organisation?

**Innovation/Individuals**

1. We are trying to consider patients’ needs to the best of our ability when creating the physical activity program that we will offer at your site/location/context [INSERT LOCATION]. From your perspective and experience is there anything else we can/should consider? (Examples: family needs, patient capabilities, patient environmental context, available resources, social influences, motivation)

**Innovation/Individuals/Outer Setting**

1. In our physical activity program that is offered in Alberta, we are assessing a range of physical and psychological outcomes before and after the physical activity program to explore its effects. I can list these for your if you’re interested. [If yes, list: the current IMPACT trial is exploring patient’s objective PA (primary effectiveness outcome) and secondary effectiveness outcomes of patient- and caregiver-reported outcomes (i.e., patient and caregiver-reported PA and patient’s quality of life, symptoms, cognitive function, resource use), and physical fitness outcomes (i.e., patient’s aerobic endurance, lower body flexibility, shoulder flexion range of motion, balance, functional mobility)].

PROBE: Based on what you know so far, what are the top outcomes you’d be interested in us including? Why? What do you think patients/parents would most want to see changed through a physical activity program?

PROBE: What would be the most convincing outcomes for us to explore from your perspective as a healthcare/allied health care provider?

PROBE: If we could only measure 3 things, what would you suggest? Why?

PROBE: What do you think the top 3 outcomes of the patients would be? Would they be similar or different? Why?

**Innovation/Individuals**

1. In our physical activity program that is offered in Alberta, patients are given a Fitbit to use throughout the study. At the same timepoints as the other assessments, baseline (week 0), post-program (week 12) and at follow-up timepoints (week 24, week 52), we ask patients to wear the Fitbit for one week and complete a tracking log to indicate how long they wore the Fitbit. We are assessing the number of steps they take at these various timepoints.

PROBE: Do you think patients and their families would be wiling/able to do this? Do you think they would prefer something else?

PROBE: What do you think your patients' families would want the device to look like or tell them?

**Innovation/Individuals**

1. In our physical activity program that is offered in Alberta, patients are also given a yoga mat that they can use during the program and are able to keep once they are done in the program.

PROBE: What is your sense/thoughts/feelings about this?

PROBE: If we were to provide patients with something to support their participation in the program as I have described it, what do you think it would be?

PROBE: Is there anything else we can give to patients that you think will increase their motivation to do physical activity?

PROBE: Is there anything else we should consider?

PROBE: I should also share that with the funding we have; we are able to provide devices (eg, iPads) with data so that those without access to an iPad or internet can still take part. What do you think of this? How do you think we should advertise this? Do you think there are other things we should include/offer to ensure as many children and adolescents can benefit as possible?

**Other**

1. Is there any other information you would like to share with us?

**Personnel at Support Organizations Semi-Structured Interview Guide**

[START AUDIO RECORDER]

Hello, my name is [INSERT NAME], and I am [INSERT ROLE WITH STUDY], it is nice to meet you. As you may recall from the informed consent that you completed, we are conducting this study to adapt an existing physical activity program, called IMPACT to [INSERT LOCATION].

Before we begin, we just want to confirm your location. Since we are adapting in BC, ON, and the Maritime provinces, we want to ensure we are tailoring the program to your location. What city are you currently located in and what support organization are you apart of?

As a first step, we want to speak with you and people in similar roles as you, as well as healthcare providers, staff, and patients/survivors and their caregivers to understand if or how we need to adapt IMPACT and what we need to consider to successfully run a study evaluating IMPACT to your location/site/context.

Essentially, the goal is for us to make sure we are considering barriers so that we can develop strategies to overcome them in advance and to identify facilitators, so we can do what we can to maximize them.

Did you have any questions for me after you read the consent form? Or question about this research overall?

[If yes, answer accordingly].

[If not, continue with questions below].

I am hoping you saw the document that was sent when this interview was scheduled. The document provides a brief overview of IMPACT and how it’s being implemented and evaluated in Alberta.

Briefly, IMPACT is a physical activity program for children and adolescents with any **cancer or blood disorder**, who are **between the ages of 5-18**, who are **awaiting-, on-, or within 3 months of completing treatment**. The physical activity program is delivered by **videoconference** using Zoom, 1:1 by a trained exercise professional **2-3 times/week over 3 months (12 weeks).** Each session is 15-45 minutes long and is tailored based on how the patient is feeling (energy, fatigue, treatment-related side-effects), their age, and their preferences.

Do you have any questions about this?

We are evaluating this program using a study design that allows us to look at things that influence implementing the program and effectiveness – so we are measuring a range of outcomes before and after the program and at 2 follow-up times to see if there’s any change. I should note that our study design also enables *all* patients to take part – there is no randomization, and this is because we know that physical activity is safe, feasible, beneficial, and recommended for pediatric cancer patients.

Do you have any questions?

Overall, what modifications or tailoring do you believe the program needs to undergo to fit within your setting? What challenges/complications associated with offering a physical activity program in [INSERT LOCATION] do you foresee?

I will now ask you some specific questions about elements of the physical activity program in Alberta and your thoughts as to what needs to change/be considered to work in your setting/community.

**Innovation**

1. So, as we move through the questions I have for you, I will first tell you what we are currently doing in Alberta and then ask for your thoughts. We will start with participants.

First, you should know that IMPACT was developed using the literature and resources we have available in pediatric cancer. However, given pragmatic nature of the program, early findings for safety of 1:1, tailored physical activity, and similarities in some treatment and thus side-effects, we decided to offer the physical activity program to children and adolescents affected by blood disorder as well. As mentioned, children and adolescents diagnosed with any cancer *or* blood disorders, who are between the ages of 5-18, who are awaiting-, on-, or within 3 months of completing treatment are eligible.

PROBE: What is your sense/thoughts/feelings about this?

PROBE: What do you think about physical activity for children and adolescents affected

by cancer and blood disorders?

PROBE: Do you think it is appropriate to include both children and adolescents affected by cancer and blood disorder in your context? Why/why not?

PROBE: When should we offer the physical program we are creating? During treatment for cancer or blood disorder? After treatment? Both? Why?

PROBE: What are your thoughts on the age range of 5-18 years? Do you believe this age range should be increased to 19-20 year olds?

PROBE: Do you have opportunities available for patients and/or survivors at your organization? Is there anything else we should consider?

**Implementation Process/Inner Setting**

1. We are recruiting participants through consent to contact forms, do you know what this is? [if not, “this form is completed by caregivers where they indicate if they are interested in receiving more information about the study and their preferred method of contact (i.e., phone call, text, email) which is then sent to the study staff who then take over communication]. Other recruitment methods include advertising (e.g., social media, emails from support organizations), and word of mouth (i.e., past participant referral).

PROBE: What is your sense/thoughts/feelings about this approach? PROBE: Are there any other recruitment methods we should consider?

PROBE: Would sending emails from your organization to potential participants/families be a feasible and acceptable recruitment method?

PROBE: Do you think your organization/team has capacity to send emails like this? Who would be the person to send those emails?

PROBE: What has worked well in the past for recruitment to trials?

PROBE: What can we do to make it easier for your team? What additional resources/documents might be needed?

PROBE: How else should families hear about the program?

1. In Alberta, over 95 children and adolescents have heard about the program, but only 15 have participated. Does your organization face similar enrollment issues or have you seen anything like this in the past?

PROBE: People are interested but then do not enroll, do you have any ideas for how we could close that gap and ensure more children and adolescents who are referred, consent and enrol?

PROBE: Do you have recruitment methods that have proven to be successful?

PROBE: What do you think we can do to attract more children, adolescents, and their caregivers, to participate? For this program we are creating, how do you think we can involve more children and adolescents?

**Implementation Process**

1. The physical activity program offered in Alberta was developed based on input from semi-structured interviews like this with healthcare providers and staff from the two children’s hospitals in Alberta and international experts (researchers, qualified exercise professionals) who deliver physical activity to children and adolescents affected by cancer. We now have a patient advisory board comprised of patients, caregivers, healthcare providers, community partners, and the study staff, who review and comment on aspects of IMPACT and the trial.

PROBE: What is your sense/thoughts/feelings about this development approach?

PROBE: Should we consider anything/anyone else?

PROBE: When we are adapting, is there a perspective we might be missing?

**Implementation Process/Innovation/Inner Setting**

1. In the physical activity program offered in Alberta, one thing we do to support safety is have healthcare providers and allied providers, like physiotherapists and nurses, provide medical clearance that it is safe for the child to engage in physical activity or they note specific/notable considerations that exercise professional delivering the sessions can take into account.

PROBE: What is your sense/thoughts/feelings about this?

PROBE: If your organization requires clearance for physical activity, or anything else, how do you do it?

PROBE: Do you envision any challenges with getting medical clearance?

PROBE: Do you think it makes sense that any healthcare or allied provider can provide clearance?

PROBE: What methods/strategies have you used for medical clearance that have been successful?

PROBE: Is there anything else we should consider?

**Implementation Process/Innovation/Inner Setting**

1. In the physical activity program in Alberta, another consideration for safety is that we ask a caregiver or adult to be present during the sessions. This could mean being in the same room, doing the activities, or another room in the house.

PROBE: What is your sense/thoughts/feelings about this?

PROBE: Is there anything else we should consider re: safety?

**Implementation Process/Innovation**

1. The physical activity program in Alberta is delivered by a trained qualified exercise professional who has experience delivering online physical activity for individuals (children, adolescents, young adults, adults) affected by cancer and have undergone study-specific training.

PROBE: What is your sense/thoughts/feelings about this?

PROBE: Would you feel comfortable promoting this study given who is delivering it?

PROBE: Is there anything else we should consider?

PROBE: Is there anyone else we should consider when delivering the program? What strategies are needed to coordinate this process?

PROBE: Is there anything you do to ensure safety in your programs that you think we should consider?

**Inner Setting**

1. Do you think your support organization would be interested and/or supportive of a physical activity program? Does supporting this program align with your organizations’ missions and goals? If yes, how? If not, describe.

PROBE: Are there people who might be opposed or unsupportive? What might we do to leverage their support? How could we gain their support?

PROBE: What kinds of changes are needed to facilitate recruiting to this program within your organization? (E.g., structural, work, IT)

PROBE: What changes need to be made to embed referral to the physical program into your organisation?

**Innovation/Individuals**

1. We are trying to consider participants’ needs to the best of our ability when creating the physical activity program we will offer at your site/location/context [INSERT LOCATION]. From your perspective and experience is there anything else we can/should consider? (Examples: family needs, patient capabilities, patient environmental context, available resources, social influences, motivation)

**Innovation/Individuals/Outer Setting**

1. In our physical activity program in Alberta, we are measuring a range of physical and psychological outcomes to see what effect, if any, the physical activity program has. I can list the outcomes we are measuring for you if you’d like. [If yes, list: the current IMPACT trial is exploring patient’s objective PA (primary effectiveness outcome) and secondary effectiveness outcomes of patient- and caregiver-reported outcomes (i.e., patient and caregiver-reported PA and patient’s quality of life, symptoms, cognitive function, resource use), and physical fitness outcomes (i.e., patient’s aerobic endurance, lower body flexibility, shoulder flexion range of motion, balance, functional mobility)].

PROBE: Based on what you know so far, what are the top outcomes you’d be interested in? Why? What do you think participants/parents would most want to see changed through a physical activity program?

PROBE: If we could only measure 3 things, what would you suggest? Why?

PROBE: What do you think the top 3 outcomes of the participants would be? Would they be similar or different? Why?

**Innovation/Individuals**

1. In the physical activity program in Alberta, participants are given a Fitbit to use throughout the study. At the same timepoints as the other assessments, baseline (week 0), post-program (week 12) and at follow-up timepoints (week 24, week 52), we ask participants to wear the Fitbit for one week and complete a tracking log to indicate how long they wore the Fitbit. We are assessing the number of steps they take at these various timepoints.

PROBE: Do you know what Fitbits are? Have you ever worn one?

PROBE: What do you think about participants wearing these at different timepoints? How can we support participants to wear this at different timepoints?

PROBE: Do you think participants’ and their families would be wiling/able to do this? Do you think they would prefer something else?

PROBE: What do you think your participants’ families would want the device to look like or tell them?

**Innovation/Individuals**

1. In Alberta, participants are also given a yoga mat that they can use during the program and are able to keep once they are done in the program.

PROBE: What is your sense/thoughts/feelings about this?

PROBE: If we were to provide participants with something to support their participation in the physical activity program as I have described it, what do you think it would be?

PROBE: Is there anything else we can give to participants that you think will increase their motivation to do physical activity?

PROBE: Is there anything else we should consider?

PROBE: I should also share that with the funding we have, we are able to provide devices (eg, iPads) with data so that those without access to an iPad or internet can still take part. What do you think of this? How do you think we should advertise this? Do you think there are other things we should include/offer to ensure as many children and adolescents can benefit as possible?

**Other**

1. Is there any other information you would like to share with us?
